# Antimicrobial resistance among *Escherichia coli *that cause childhood community-acquired urinary tract infections in Northern Italy

**DOI:** 10.1186/1824-7288-37-3

**Published:** 2011-01-06

**Authors:** Alessandra Caracciolo, Alberto Bettinelli, Claudio Bonato, Clementina Isimbaldi, Alessandro Tagliabue, Laura Longoni, Mario G Bianchetti

**Affiliations:** 1 Department of Clinical Laboratory, San Leopoldo Mandic Hospital, Merate-Lecco, Italy; 2 Department of Pediatrics, San Leopoldo Mandic Hospital, Merate-Lecco, Italy; 3 Department of Pediatrics, Mendrisio and Bellinzona Hospitals, Switzerland, and University of Bern, Switzerland

## Abstract

**Abstracts:**

**Background**

Resistance rate of *Escherichia coli *against antimicrobials that are commonly prescribed in pediatric urinary tract infections is currently a matter of concern.

**Methods**

The antimicrobial susceptibility patterns of uropathogenic *Escherichia coli *strains to the common antibimcrobials ampicillin, cotrimoxazole, coamoxyclav, ceftazidime, ceftriaxone, nitrofurantoin, and gentamycin were determined in 177 children aged from 2 to 36 months. They presented with their first symptomatic community acquired urinary tract infection at the Department of Pediatrics, San Leopoldo Mandic Hospital, Merate-Lecco.

**Results**

High rates of ampicillin (inpatients: 50%; outpatients: 52%) resistance were identified. The resistance for cotrimoxazole (inpatients: 22%; outpatients: 15%) and especially coamoxyclav (inpatients: 6%; outpatients: 10%) was less pronounced than that to ampicillin. No resistance or less than 1% of resistance was identified for ceftazidime, ceftriaxone, nitrofurantoin, and gentamycin both in inpatients and in outpatients.

**Conclusions**

Italian children affected with a community acquired urinary tract infection are initially managed orally with coamoxyclav or parenterally with ceftriaxone. The results of the present retrospective analysis support this attitude. Parenteral ceftriaxone or an aminoglycoside should be considered for patients on antimicrobial prophylaxis or recently prescribed antimicrobials.

## Background

Urinary tract infections are common bacterial infections of childhood [1,2]. Prompt recognition and correct antimicrobial management relieve symptoms and prevent kidney damage [1,2]. Since drug management is usually started before the responsible microorganism and its susceptibility to antimicrobial agents are known, the choice relies on local epidemiologic data. Considering that *Escherichia coli *accounts for 60 to 80% of community acquired urinary tract infections, initial empiric therapy includes antimicrobial agents that provide coverage for that particular bacterium [1,2].

The results of a study performed between 2000 and 2005 in North East Italy indicate that both oral coamoxyclav and parenteral ceftriaxone are very effective in the management of childhood pyelonephritis [3]. Resistance to commonly prescribed antimicrobials agents, caused among others by the production of extended spectrum beta lactamases, is currently a matter of increasing concern [4]. We studied the susceptibility to antimicrobials of *Escherichia coli *strains that cause symptomatic community-acquired urinary tract infections in small children treated at our institution.

## Materials and methods

The study is a retrospective analysis of bacteria isolated from urinary tract cultures performed during the 3-year period from 2007 to 2009 at the Department of Pediatrics, San Leopoldo Mandic Hospital, Merate (Lecco), a Northern Italy hospital.

We included exclusively children aged from 2 to 36 months presenting with their first symptomatic community acquired urinary tract infection. Infants less than 6 months of age, toxic or ill appearing children, and patients unable to retain oral fluids were admitted for parenteral antimicrobial management. Uncertainty about outpatient adherence to the recommended regimen of care was a further indication for admission. Children with known urinary tract malformations or antimicrobial prophylaxis were excluded. In the patients urine, collected either by clean catch method or by bladder catheterization, contained 25 or more white cells per μicroliter, and the culture grew only one micro-organism and ≥ 100,000 colony forming units per milliliter (midstream collection) respectively ≥ 10,000 colony forming units per milliliter (transurethral catheterization).

Standard techniques were used for culture and identification of pathogens [5]. *Escherichia coli *susceptibility testing was performed using the disk diffusion technique according to the guidelines of the Clinical and Laboratory Standards Institute. Inhibition halo diameter was interpreted for each isolate to determine susceptibility, which was defined as susceptible, intermediate, or resistant [5]. Following common antimicrobials were tested: ampicillin, coamoxyclav, cotrimoxazole, ceftazidime, ceftriaxone, nitrofurantoin, and gentamycin.

For analysis the two-tailed Fisher exact test was used [6]. Statistical significance was defined as a P value of < 0.05.

## Results

During the study period, a total of 275 consecutive children, 145 (53%) girls and 130 (47%) boys between 2 and 36 months of age, were included. Urine had been obtained by clean catch method in 243 and by bladder catheterization in the remaining 32 children. Germs other than *Escherichia coli *caused the infection in 98 (36%) cases: *Proteus mirabilis *(N = 40; 15%), *Pseudomonas aeruginosa *(N = 19; 7%), Enterococcus species (N = 17; 6%), *Klebsiella pneumoniae *(N = 12; 4%), other uropathogens (N = 10; 4%).

*Escherichia coli *was isolated in the remaining 177 (64%) children. This germ was isolated in 153 of the 243 (63%) collections performed by clean catch method and in 24 of the 32 (75%) collections performed by bladder catheterization (difference not significant).

**Figure 1:**
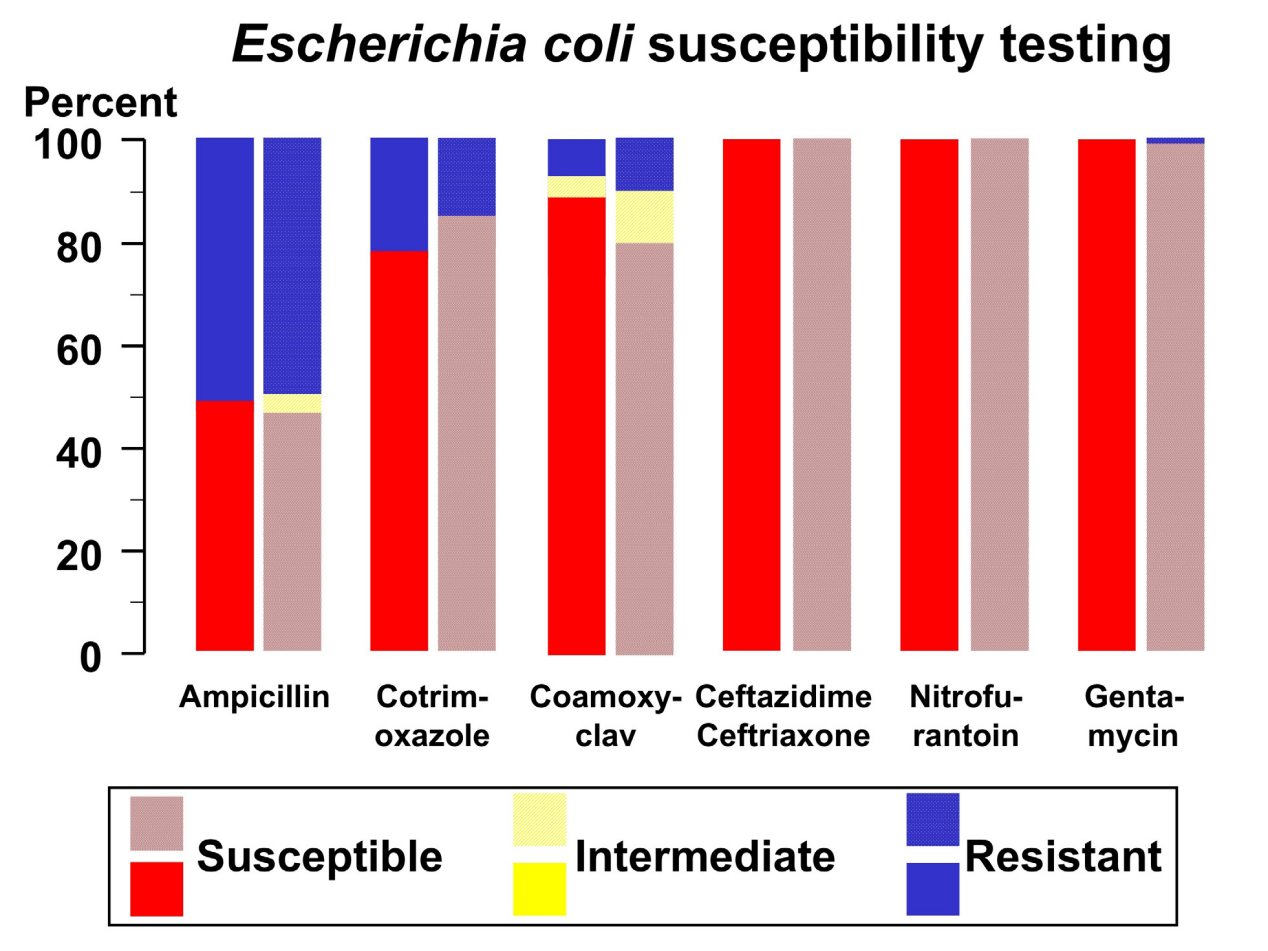
**Susceptibility to ampicillin, cotrimoxazole, coamoxyclav, ceftazidime, ceftriaxone, nitrofurantoin, and gentamycin in 177 pediatric urinary tract infections caused by *Escherichia coli***. The affected children were treated either as inpatients (N = 68; 38%) or as outpatients (N = 109; 62%). Inpatients are given full colors, outpatient patterned colors. No difference was noted between inpatients and outpatients.

The antimicrobial susceptibility of isolated *Escherichia coli *strains appears in figure 1. Considering that uropathogenic *Escherichia coli *strains with intermediate susceptibility are considered clinically sensitive, high rates of ampicillin (inpatients: 34 cases, 50%; outpatients: 57 cases, 52%) resistance were identified. The resistance for cotrimoxazole (inpatients: 15 cases, 22%; outpatients: 16 cases, 15%) and especially coamoxyclav (inpatients: 4 cases, 6%; outpatients: 11 cases; 10%) was less pronounced than that to ampicillin. No resistance or less than 1% of resistance was identified for ceftazidime, ceftriaxone, nitrofurantoin, and gentamycin both in inpatients and in outpatients. The resistance rate was not statistically different in inpatients as compared with outpatients.

Age distribution ranged between 2 and 12 months in 107 (60%) and between 13 and 36 months in the remaining 70 subjects (40%), as given in table 1. While the majority of children aged between 2 and 12 months were admitted, the majority of those aged between 13 and 36 months were not admitted (P < 0.001). Furthermore, while the majority of the children aged between 2 and 13 months were boys, the majority of those aged between 13 and 36 months were girls (P < 0.001). The antimicrobial susceptibility of isolated *Escherichia coli *strains was similar in both age groups.

**Table 1:** Influence of age on resistance to ampicillin, cotrimoxazole, and coamoxyclav in 177 pediatric urinary tract infections caused by Escherichia coli

	2-12 Months of Age	13-36 Months of Age	Significance
N	107	70	
Gender, male: female	57: 50	19: 51	P < 0.001
Inpatients/outpatients	50/57	18/52	P < 0.001
Escherichia coli Resistance, N (%)			
Ampicillin significant	56 (52%)	35 (50%)	Not
Coamoxyclav significant	6 (6%)	9 (13%)	Not
Cotrimoxazole significant	16 (15%)	15 (21%)	Not

No resistance or less than 1% of resistance was identified for ceftazidime, ceftriaxone, nitrofurantoin, and gentamycin.

## Discussion

The results of the present analysis performed in small children with their first *Escherichia coli *community acquired urinary tract infection confirm that these infections are more prevalent among males during the first year of life and among females thereafter [1,2]. Furthermore our data confirm that the resistance rate of *Escherichia coli *against cotrimoxazole and especially ampicillin, antimicrobials that are commonly prescribed in these children, is currently a matter of concern. On the contrary, resistance against coamoxyclav was uncommon. Finally, no significant resistance was noted against nitrofurantoin, third-generation cephalosporins like ceftazidime or ceftriaxone, and the established aminoglycoside gentamycin.

Antimicrobial resistance has reached crisis stage in human medicine [4]. The crucial factor underlying resistance is antimicrobial use within both human and veterinary medicine (the greater the duration of exposure, the greater the risk of the development of resistance irrespective of the severity of the need for antimicrobials). In the present analysis children on antimicrobial prophylaxis and patients with hospital acquired infections were not included: in these patients testing for resistance is biased towards higher rates of resistance [4]. However, we did not specifically address the possible prescription of antimicrobials in the weeks preceding the urinary tract infection, a further possible cause of reduced antimicrobial susceptibility [1,2].

In this study *Escherichia coli *was isolated more frequently in urine obtained by clean catch method than in urine obtained by bladder catheterization. This observation might suggest some degree of contamination in samples obtained without catheterization.

High resistance rates of *Escherichia coli *against ampicillin and cotrimoxazole have also been recently noted in various European countries, including Switzerland and Austria, indicating that management with these agents may be inadequate in many cases [7-9].

Susceptibility to nitrofurantoin has remained practically unchanged since its introduction into clinical practice more than 50 years ago [10]. Since standard medication with nitrofurantoin does not achieve therapeutic concentrations in the bloodstream, its use is advised only for uncomplicated cystitis [10]. Fluoroquinolones are widely prescribed to treat urinary tract infections in adulthood [11]. We did not address the susceptibility pattern of these drugs to *Escherichia coli *because studies in juvenile laboratory animals suggest there may be an increased risk of fluoroquinolone-associated cartilage lesions [11].

Coamoxyclav is currently widely used and recommended [12] in Italy for the empirical oral management of children affected with a community acquired urinary tract infection. The rate of in vitro resistance to this antimicrobial noted in the present study performed between 2007 and 2009 is almost identical to that noted in North East in Italy between 2000 and 2005 [3]. Considering that coamoxyclav is concentrated in the urine and susceptibility testing is mostly based upon blood concentration determinations, this antimicrobial might often be effective for the treatment of urinary tract infections even when in vitro susceptibility testing suggests full resistance. On the other side, the very good susceptibility pattern of isolated Escherichia coli to third-generation cephalosporins and aminoglycosides support the current role of these drugs in the treatment of childhood urinary tract infection.

In this study, the susceptibility to antimicrobials was identical and age-independent in inpatients and outpatients with a community acquired urinary tract infection, as previously noted in the literature [1,2]. These observations suggest, that in uropathogens bacterial virulence that causes severe disease and, subsequently, increases the risk factor for hospitalization, is not related to susceptibility to antimicrobials.

## Conclusions

The results of the present analysis support the recommendations of the Italian Society of Pediatric Nephrology to initially manage children affected with a community acquired urinary tract infection with either oral coamoxyclav or parenteral ceftriaxone. Parenteral ceftriaxone or an aminoglycoside should be considered for patients on antimicrobial prophylaxis or recently prescribed antimicrobials.

## Competing interests

The authors declare that they have no competing interests.

## Authors' contributions

AC, AB and LL drafted the manuscript. CB, CI and AT participated in the design of the study. MGB coordinated the study and performed the figure and the statistical analysis. All authors read and approved the final manuscript.

## References

[B1] Chang SL Shortliffe LD Pediatric urinary tract infections Pediatr Clin North Am 2006 53 379 400 10.1016/j.pcl.2006.02.011 16716786

[B2] Hellerstein S Acute urinary tract infection - evaluation and treatment Curr Opin Pediatr 2006 18 134 138 10.1097/01.mop.0000193271.09001.a3 16601492

[B3] Montini G Toffolo A Zucchetta P Dall'Amico R Gobber D Calderan A Maschio F Pavanello L Molinari PP Scorrano D Zanchetta S Cassar W Brisotto P Corsini A Sartori S Da Dalt L Murer L Zacchello G Antibiotic treatment for pyelonephritis in children: multicentre randomised controlled non-inferiority trial BMJ 2007 335 386 388 10.1136/bmj.39244.692442.55 17611232 PMC1955287

[B4] Paschke AA Zaotius T Conway PH Xie D Keren R Previous antimicrobical exposure is associated with drug-resistant urinary tract infections in children Pediatrics 2010 125 664 672 10.1542/peds.2009-1527 20194282

[B5] Holland TL Woods CW Joyce M Antibacterial susceptibility testing in the clinical laboratory Infect Dis Clin North Am 2009 23 757 790 10.1016/j.idc.2009.06.001 19909884

[B6] Brown GW Hayden GF Nonparametric methods. Clinical applications Clin Pediatr (Phila) 1985 24 490 498 10.1177/000992288502400905 4017399

[B7] Borsari AG Bucher B Brazzola P Simonetti GD Dolina M Bianchetti MG Susceptibility of Escherichia coli strains isolated from outpatient children with community-acquired urinary tract infection in Southern Switzerland Clin Ther 2008 30 2090 2095 10.1016/j.clinthera.2008.11.002 19108796

[B8] Fritzsche M Ammann RA Droz S Bianchetti MG Aebi C Changes in antimicrobial resistance of Escherichia coli causing urinary tract infections in hospitalized children Eur J Microbiol Infect Dis 2005 24 233 235 10.1007/s10096-005-1301-2 15772820

[B9] Prelog M Schiefecker D Fille M Wurzner R Brunner A Zimmerhackl LB Febrile urinary tract infection in children: ampicillin and trimethoprim insufficient as empirical mono-therapy Pediatr Nephrol 2008 23 597 602 10.1007/s00467-007-0701-1 18193296

[B10] Cunha BA New uses for older antibiotics: nitrofurantoin, amikacin, colistin, polymyxin B, doxycycline, and minocycline revisited Med Clin North Am 2006 90 1089 1107 10.1016/j.mcna.2006.07.006 17116438

[B11] Koyle MA Barqawi A Wild J Passamaneck M Furness PD Pediatric urinary tract infections: the role of fluoroquinolones Pediatr Infect Dis J 2003 22 1133 1137 10.1097/01.inf.0000101849.11912.8e 14688587

[B12] Montini G Ammenti L Cataldi L Chimenz R Fanos V La Manna A Marra G Materassi M Pecile P Pennesi M Pisanello L Sica F Toffolo A Le infezioni febbrili delle vie urinarie Medico e Bambino 2009 28 359 370

